# Marketing for Special and Academic Libraries: A Planning and Best Practices Sourcebook

**DOI:** 10.5195/jmla.2017.116

**Published:** 2017-01

**Authors:** Marian T. Simonson

Kudos to Valerie St. Pierre Gordon, AHIP, and Patricia E. Higginbottom for writing such a fabulous book on marketing for special and academic libraries. This title is based on their research and practical experience working at the University of Alabama at Birmingham’s Lister Hill Library of the Health Sciences. The book’s premise is simple: libraries need to demonstrate their value to administration to justify expenditures on resources. Libraries also need to communicate their value to their customers to encourage them to utilize these resources. This is a daunting task for librarians who already have too much to do and have received little to no training in marketing.

For all of us in this position, help is here in this gem of a book. Chapter one starts with a discussion of the importance of a strategic plan and provides an outline to create one. In Gordon and Higginbottom’s view, the strategic plan is the pivotal tool to use in developing and implementing a marketing plan. Chapters two to five address specific components of the marketing plan, whereas chapters six to ten discuss specific marketing modalities. The book concludes with a sample marketing plan and web usability test in the two appendixes.

The text is easy to read, and the concepts are well illustrated with examples, case studies, figures, and tables. For those of us who want to delve further into any aspect of marketing, the authors provide references to books, journal articles, and websites that they have personally vetted. Enough information is provided in each chapter to allow any librarian to jump right into marketing without feeling overwhelmed. There are important take-home points on each page, and chapters one to five build on each other, so I recommend reading the book cover-to-cover. On the other hand, those who like to read in a nonlinear fashion will be able to follow the text due to the clarity of the writing.

As I read this book, I identified with many of the points and felt that the book was written specifically for me. The phrase “just in time” marketing is an apt description. I especially appreciate the authors’ willingness to share mistakes and failures so that readers can all benefit from their experience.

I highly recommend this book to any librarians who are interested in getting to the crux of marketing their library, staff, and resources in an efficient and effective manner. Ideas are presented for health sciences librarians in academic, hospital, and special libraries. Librarians in specialties other than health sciences will also benefit because it is easy to apply the ideas in this book to other subjects.

